# The influence of policy advocacy and education on medical staff’s adaptation to diagnosis related groups payment reform in China: an analysis of the mediating effect of policy cognition

**DOI:** 10.3389/fpubh.2024.1375739

**Published:** 2024-11-13

**Authors:** Zhi-Ying Ni, Bo-Kai Zhang, Lei Song, Zhao-Yan Zang, Hong Yu

**Affiliations:** Sir Run Run Shaw Hospital, Zhejiang University School of Medicine, Hangzhou, China

**Keywords:** DRGs payment reform, policy advocacy and education, policy cognition, adaptation, mediating effect

## Abstract

**Introduction:**

In recent years, China has been carrying out the Diagnosis Related Groups (DRGs) payment reform, which has an impact not only on payment methods and medical expenses, but also on the behaviors of medical staff. Some of these behaviors are unexpected by policymakers, such as turning away critically ill patients, disaggregating hospitalization costs, setting up disease groups with higher points, and so on. This phenomenon attracted the attention of some scholars, who put forward a few positive intervention measures, mainly including policy advocacy and system improvement. The scholars believed that the former was more feasible. However, there is a lack of research on the effects and influencing processes of these interventions. Therefore, this study aims to explore the influence of policy advocacy and education on medical staff’s adaptation to DRGs payment reform in China and the role of policy cognition in this process, in order to provide experiences for the smooth implementation and sustainable development of DRGs payment system.

**Methods:**

A questionnaire survey was conducted among 650 medical staff in five large general hospitals in Zhejiang Province, China, to understand their participation and feedback on policy advocacy and education, their adaptation to the current DRGs payment reform, and their cognition of relevant policies. After descriptive statistical analysis, partial correlation analysis, multiple linear regression models and bias correction Bootstrap sampling method were used to analyze the mediating effect of policy cognition factors.

**Results:**

All respondents had participated in organized collective policy advocacy and education activities in the past year, but the degree of satisfaction and recognition was not very high. 59.5 percent said their adaptation to the DRGs payment reform was average. Nearly half did not regularly pay attention to and participate in the management of the medical costs of patients with DRGs through compliance. And they had a low understanding of the specific rules of DRGs payment and did not form a high policy identity. The mediating effect values of policy cognition were 0.148, 0.152, 0.108, and 0.057, respectively, when the frequency and quality of policy advocacy and education influenced medical staff’s adaptive perception and adaptive behaviors.

**Discussion:**

The organized collective policy advocacy and education can promote medical staff’s adaptation to DRGs payment reform by improving their policy cognition, and the action paths are diverse. Policymakers and hospital managers need pay attention to this phenomenon, and formulate demand-centered, value-oriented whole-process advocacy and education strategies while constantly improving the DRGs payment system. All of these provided a basis for further research and practice of positive intervention in DRGs payment reform.

## 1 Introduction

### 1.1 The origin and development of DRGs

Diagnosis Related Groups is a tool to measure the quality and efficiency of medical services or measure the amount paid by the health insurance fund. It is a case cluster statistical method for hospital management, considering the diagnosis of disease, treatment, complications, comorbidities, as well as the age of patients, resource consumption, and other factors (1, 2). Diagnosis Related Groups-Prospective Payment System (DRG-PPS) is to apply the above method to group the cases, in advance to establish the payment standard for each patient group, according to which the medical expenses are paid. This is a new medical insurance fund payment method that is different from the traditional payment by service item or capitation (3). DRGs was first developed by Yale University in 1969. After many practices and adjustments proved that its fee control effect was significant, and the national uniform rate standard was achieved in 1987, so far, several versions of Medicare Severity-Diagnosis Related Groups (MS-DRG) have been issued (4, 5). Since then, Australia, Germany, France, Japan, and other developed countries in order to control the rise of medical costs and improve the efficiency of medical services, have explored the implementation of a DRGs payment system and made a series of adjustments according to their respective situations, and achieved good results (6–8). At the same time, the reform of DRGs had also promoted the unification of their domestic International Classification of Diseases (ICD) versions, medical service prices, and medical insurance payment standards, and strengthened their assessment and supervision of medical institutions (9–11). However, there were also some problems in this process, such as the unreasonable disease grouping, the insufficiently friendly compensation mechanism of new clinical technology, the emergence of negative behaviors of doctors, and so on (12–14).

### 1.2 DRGs payment reform in China

In recent years, in order to deal with the chain problems of the aging population, excessive medical treatment, rapid growth of medical expenditure, and increasing pressure on medical insurance funds, many developing countries have also begun to reform the DRGs payment system (15). Among them, China, as a populous country, the above problems are particularly severe, and it is urgent to reform the payment method of medical insurance (16, 17). In fact, as early as the 1980s, China began to explore the feasibility of implementing DRGs at the theoretical level. In 2008, the construction of the first DRGs grouping device in the Chinese Mainland was completed, and from 2011 to 2017, the DRGs payment reform pilot work was carried out in Beijing, Shenzhen, Sanming, and other places, during which multiple DRGs grouping schemes were used, including BJ-DRG, CN-DRG, and C-DRG (2, 18). Since the establishment of the National Healthcare Security Administration in 2018, the national general grouping standard (China Healthcare Security-Diagnosis Related Groups, CHS-DRG) has been formulated based on the previous different grouping schemes, and the pilot scope of reform has been continuously expanded, requiring all eligible medical institutions to complete the reforms related to DRGs payment method by the end of 2025 (19). It is worth noting that in September 2020, Zhejiang Province became the first province in China except Taiwan Province to implement DRGs payment reform in the whole province, and it had its characteristic grouping scheme (ZJ-DRG), and was currently in a critical period of transition to CHS-DRG (20).

### 1.3 Research questions and objectives

Based on relevant studies and reports, we found that although China’s DRGs payment reform had played a certain role in fee control and improved the efficiency of medical services, it had also brought about a series of problems (10). These problems were mainly manifested in the medical staff in the process of reform to turn away critically ill patients, disaggregate hospitalization costs, discharge the patient early, set up disease groups with higher points, and other unexpected behaviors (13, 21). Although these behaviors were considered to be the reasonable feedback of stakeholders’ mutual game under the combined influence of reform enforcement and system defects (13). However, these behaviors are contrary to the goals of the reform and are non-compliant fee control behaviors. If there is no effective intervention, it will not only hinder the sustainable development of the reform but also affect the quality of medical services. From the point of view of system theory and social ecology, medical staff with such adverse stress reactions had not adapted to the changes in the current medical insurance payment system, and they were not coordinated with the operation of the new system (22, 23). Without proper control and guidance, they could affect the balance and development of the system. Therefore, even though there are still some defects in the new payment system, the mandatory and dynamic development of the reform is undeniable, so how to improve the adaptation of stakeholders to the complex system is the key issue.

At present, more and more scholars have begun to pay attention to the impact of DRGs payment reform on medical staff, and their research has gradually shifted from macro policy evaluation to micro policy impact (13, 21, 24). Most of these studies focused on the investigation of cognition and behavior and explored the relationship between the two. Among them, in addition to improving the defects of the system itself, some active intervention measures had been proposed, among which the priority of policy advocacy and education was higher, but there was a lack of further research on its effect and action process (25, 26). To sum up, this study aims to explore the influence of policy advocacy and education on adaptation to DRGs payment reform and its action process from the perspective of medical staff combining multidisciplinary theories. Based on this, we can propose some more scientific and effective action plans, which will not only make up for the shortcomings of relevant research fields but also provide a basis for those countries or regions that are undergoing DRGs payment reform to formulate effective interventions to promote the smooth implementation and sustainable development of the new payment system.

## 2 Theoretical basis and research hypothesis

The word “adaptation” first appeared in Darwin’s “Origin of Species,” his theory of evolution pointed out “natural selection, survival of the fittest,” emphasizing that adapting to environmental changes is the nature of organisms (27). Later, ecosystem theory and ecological balance theory specified the importance of adapting to the environment and harmonious coexistence, and the relationship between individuals and the environment will not only affect the survival of individuals, but also affect the sustainable development of the whole system (28). The derived social ecosystem theory divided the interaction between individual development and the social environment into three major systems: micro, meso, and macro (29). These theories illustrated the importance and complexity of the relationship between human beings and social environment. With the concept of “social adaptation” put forward by Herbert Spencer, it was clear that it was a process of an individual’s response to the social environmental stimuli in line with norms, and finally reached the state of accepting the existing social norms, including both psychological and behavioral dimensions (30). Subsequently, many scholars focused on the measurement and intervention of people’s social adaptability, especially in the fields of social support and health education (31–33). However, there was no unified concept or scale of adaptation to policy reform, so this study combined Merton’s adaptation typology and Yanyan Chi’s research on the adaptability of rural disabled people to medical security and considered the mandatory and binding nature of DRGs payment reform (34, 35), it is believed that the real ideal state of adaptation should be that the goals and contents of autonomous actions meet the requirements of reform, that is, medical staff take the initiative to achieve the goal of cost control and efficiency improvement through compliance.

According to the above definition of adaptation state in the face of reform, this is not only the behavior manifestation of policy internalization at the highest level of policy acceptance response in public policy science, but also the final internalization state in Kelman’s three-process theory of attitude change (36, 37). It can be seen that good policy awareness and policy identity which can be collectively referred to as policy cognition are important factors affecting whether the adaptation to reform can be successfully achieved (38). This is also consistent with the knowledge-attitude-practice theory on the process of people’s behavior change (39). Then, policy advocacy and education is precisely considered as an information intervention means for policymakers to improve the compliance and implementation effect of policies and improve the awareness and identity of policies by target groups through various ways (40, 41). The advantage of this method is that, on the one hand, compared with punishment, threat, and other means, it can induce spontaneous behavior changes by changing people’s cognition, which is a non-coercive long-term intervention at the psychological level, and can reduce the subordination or rebellion of policy implementors (42, 43). On the other hand, compared with all kinds of regulatory measures, it belongs to the pre-intervention in the early stage of policy implementation, which has better controllability and higher cost performance (44). Long-term practice and research results have also verified the feasibility and importance of policy advocacy and education, which can help people quickly and correctly understand policies, reduce the resistance in the process of policy implementation, and contribute to the development and improvement of policies (40, 44, 45). However, in order to reduce the confusion of the research and facilitate the investigation, the policy advocacy and education in this study specifically referred to the organized collective training, lectures, or meetings on the DRGs payment system that medical staff participate in, excluding informal or personal information acquisition forms, such as through friends, personal browsing of the web, reading posters, reading books, etc.

**Figure 1:**
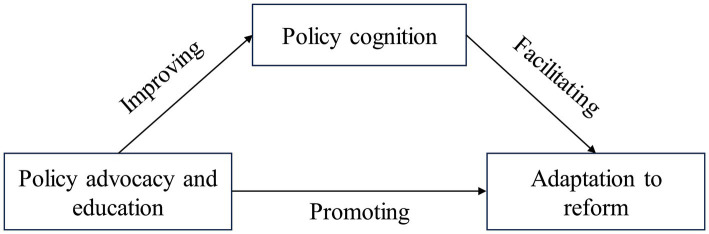
Mediation effect hypothetical path diagram.

Based on the above related theories and concepts, this study put forward the following three hypotheses, the relationship of which is shown in Figure 1. Among them, policy cognition was the intermediary variable of policy advocacy and education influencing adaptation to reform.

H1: Policy advocacy and education could promote medical staff’s adaptation to the DRGs payment reform.

H2: Policy advocacy and education could improve medical staff’s cognition of DRGs payment policy.

H3: Policy advocacy and education could help medical staff adapt to DGRs payment reform by improving their policy cognition.

## 3 Materials and methods

### 3.1 Respondents

Considering the representativeness and feasibility of the questionnaire survey, this study selected medical staff from five large Grade III and A hospitals in Hangzhou, Zhejiang Province as the respondents. In particular, it should be noted that the number of medical staff in the five hospitals ranked among the top 10 in the province, and they have implemented the DRGs payment reform earlier and more standardized. Meanwhile, their medical insurance offices were relatively mature and have conducted a lot of medical insurance policy advocacy and education.

### 3.2 Survey methods

This study collected data through field questionnaire survey. Firstly, we reasonably estimated the sample size and proportion of each hospital, occupational type (doctors, nurses, and medical technicians) and clinical department according to the difference in the number of medical staff in each hospital and the distribution of their occupational types and departments. Then experienced investigators carried questionnaires to each department for convenient sampling investigation according to the calculated quantity distribution. Meanwhile, to ensure the timeliness and accuracy of the survey results, the survey was conducted from October 15 to 30, 2023, and each questionnaire was filled out independently under the supervision of the investigator. In addition, to improve the efficiency of survey, the questionnaire was made into an electronic two-dimensional code and filled in by scanning the code on site. Finally, we sent out a total of 650 questionnaires. After data entry and elimination, the effective response rate was 97%. For details on the data collection and processing program, see the Supplementary File 1.

### 3.3 Questionnaire design

Based on the above hypothesis, this study combined literature research, case analysis, expert consultation and brainstorming methods to design the questionnaire. Besides the basic information of the respondents, it mainly included three parts: DRGs payment policy advocacy and education for medical staff, their adaptation to DRGs payment reform, and their cognition of the policy. The specific survey contents were detailed in the questionnaire section of the Supplementary File 2.

The survey of policy advocacy and education was divided into two parts: quantity and quality. The quantity was represented by the number of participants, and the quality was reflected by the feedback evaluation of the participants. It was worth noting that for the need for variable substitution in sensitivity analysis, this study set up two similar questions (recognition and satisfaction) for feedback evaluation surveys. In addition, this study also conducted a supplementary investigation on the access of medical staff to policy information and the need for policy advocacy and education content.

The survey of adaptation was also divided into two parts: self-perception (varying degrees from low to high) and objective behaviors (varying frequencies from low to high). The latter was further divided into medical behaviors (mainly including standardizing diagnosis and treatment) and non-medical management behaviors (such as standardizing the writing of medical records, analyzing cost data, learning medical insurance policies, and improving hospital operations) according to the above definition of adaptive behaviors.

The survey of policy cognition was divided into two dimensions: policy awareness and policy identity. Each dimension included multiple questions from different perspectives, and scores 1–5 according to the degree.

### 3.4 Statistical methods

#### 3.4.1 Variables processing and pre-test

In this study, the minimum dimension data containing more than two variables in the questionnaire were tested for reliability and validity. In the reliability test, the overall standardized Cronbach’s alpha was 0.89, indicating good reliability, and the Component Indicator of Total Credibility (CITC) value of each variable was greater than 0.4, indicating good correlation of variables in the same dimension. In the validity test, the Kaiser-Meyer-Olkin (KMO) value was greater than 0.9, and the Bartlett test was passed (*p* < 0.05). Meanwhile, four common factors were given, which was consistent with the questionnaire design, and the cumulative variance explanation rate after rotation was 76%, indicating that the questionnaire validity was good. On this basis, the variables (especially categorical variables) were named, assigned, and consolidated, to be included in the following statistical analysis, as shown in Table 1.

**Table 1:** Variables naming and assignment.

Types of variables	Name of variables	Variables	Variables processing
Explained variables	Y_1_	The self-perception of adaptation to reform	Assign values 1–5 in order of rank from low to high.
	Y_2_	The adaptive behaviors to reform	The mean value was calculated after the variables of the same dimension were assigned 1–4 by frequency from low to high.
Explaining variables	X_1_	The quantity of policy advocacy and education	Assign values 1–5 in order of rank from low to high.
	X_2_	The recognition of policy advocacy and education	Assign values 1–5 in order of rank from low to high.
	X_3_ (Used for sensitivity analysis)	The satisfaction with policy advocacy and education	Assign values 1–5 in order of rank from low to high.
Mediator variables	M_1_	Policy awareness	The mean value was calculated for six variables of the same dimension.
	M_2_	Policy identity	The mean value was calculated for eight variables of the same dimension.
Control variables	Control	Sex	Male = 1, Female = 2
		Age	It was actually a continuous variable.
		Education	Assign values 1–3 in order of rank from low to high.
		Occupation type	Doctor = 1, Medical technician or nurse = 2
		Positional title	Assign values 1–3 in order of rank from low to high.
		Years of service	It was actually a continuous variable.

Then, the correlation between the core variables except the basic information was verified by partial correlation analysis, which was the premise of the mediating effect test. At the same time, they were incorporated into the multiple linear regression model, and the validity and collinearity of the model were explored by the F test and Variance Inflation Factor (VIF) value. Finally, to improve the accuracy of the research results, the mediation effect test was carried out after all variables were standardized.

#### 3.4.2 Models design

According to the research hypothesis and variable processing results, the following mediation effect test models were designed.

Total effect test:

Y_1_ = β_0_ + β_1_X_1_ + β_2_X_2_ + β_3_Control + *ε* Model 1.
Y_2_ = β_0_ + β_1_X_1_ + β_2_X_2_ + β_3_Control + *ε* Model 2.

Intermediate path test:

M1 = β0 + β1X1 + β2X2 + β3Control + ε Model 3.
M2 = β0 + β1X1 + β2X2 + β3M1 + β4Control + ε Model 4.

Direct effect and intermediate effect test:

Y1 = β0 + β1X1 + β2X2 + β3M1 + β4M2 + β5Control + ε Model 5.
Y2 = β0 + β1X1 + β2X2 + β3M1 + β4M2 + β5Control + ε Model 6.

In the above models, β_0_, Control, and ε represented the constant term, the set of control variables, and the random error term, respectively, and β_1_–β_5_ were regression coefficients. Under standardized conditions, β_1_ and β_2_ in Model 1 and Model 2 represented the total effect value, β_1_ and β_2_ in Model 3 and Model 4 represented the influence of independent variables on intermediary variables, β_3_ in Model 4 represented the influence of intermediary variables M_1_ on M_2_, β_1_ and β_2_ in Model 5 and Model 6 were the direct effect values, and the cross-multiplicative values of β_1_–β_4_ in Model 3–6 were the intermediary effect values of different paths.

#### 3.4.3 Bootstrap sampling method

The Bootstrap sampling method was first proposed by Efron in 1977. It is a method to infer the population distribution of complex statistics by using the resampling technique with retracting (46). Meanwhile, it tests the significance of the intermediary effect value based on the results of multiple linear regression analysis. Compared with the traditional hierarchical regression method and the later Sobel test method, it has a higher test efficiency, which neither covers up the real intermediary effect nor is limited by the type of data distribution (47, 48). In order to further improve the accuracy and stability of research results, this study used the SPSS PROCESS tool to conduct Bootstrap sampling (5,000 times) to test the mediation effect.

## 4 Results

### 4.1 Basic information

As shown in Table 2, among the 630 valid survey subjects, the ratio of male to female was about 1:2, most of them were under 40 years old, 48.4% were doctors, half of them had obtained master’s or doctoral degrees, and 59.4% had obtained intermediate or above professional titles.

**Table 2:** The basic information of respondents.

Variables	*N*	%	Variables	*N*	%
Sex			Years of service		
Male	218	34.6	≤4	216	34.3
Female	412	65.4	5–9	158	25.1
Age			10–14	97	15.4
20–29	203	32.2	≥15	159	25.2
30–39	277	44.0	Positional title		
40–49	124	19.7	Senior	83	13.2
≥50	26	4.1	Intermediate	291	46.2
Education			Primary or below	256	40.6
Doctor’s degree	87	13.8	Occupation type		
Master’s degree	228	36.2	Doctor	305	48.4
Bachelor’s degree or below	315	50.0	Medical technician or nurse	325	51.6

### 4.2 Policy advocacy and education about DRGs payment

As shown in Table 3, all respondents had participated in organized formal advocacy and education activities on DRGs payment policies in the past year, but only about 60% of them found them helpful or satisfactory. In addition, we also found through the investigation that medical staff mainly obtained policy information through the intensive training, lectures or meetings organized by the hospital medical insurance office or superior departments, but less obtained policy information through friends, browsing the web, looking at posters, reading books, and other ways. In terms of advocacy and education content, they had the greatest demand for basic policy knowledge and the use of cost management software. In terms of the number of advocacy and education, they suggested once every quarter or half a year, not too frequently.

**Table 3:** The policy advocacy and education for medical staff.

Variables	*N*	%
The quantity of policy advocacy and education		
How many times have you received organized advocacy and education on the DRGs payment policy in the past year? (Informal or individual learning activities are not included)		
1	89	14.1
2	195	30.9
3	182	28.9
4	76	12.1
≥5	88	14.0
The quality of policy advocacy and education (Feedback and evaluation)		
Do you think it is helpful for you to understand and adapt to the DRGs payment reform after attending the policy advocacy and education?		
Not helpful at all	6	0.9
Less helpful	20	3.2
Average	226	35.9
Relatively helpful	300	47.6
Very helpful	78	12.4
What is your overall satisfaction with the policy advocacy and education you have participated in?		
Very dissatisfied	5	0.8
Somewhat dissatisfied	15	2.4
Average	288	45.7
Somewhat satisfied	262	41.6
Very satisfied	60	9.5

### 4.3 Adaptation to the DRGs payment reform

As shown in Table 4, 59.5 percent said their adaptation to the DRGs payment reform was average. Nearly half did not regularly pay attention to and participate in the management of the medical costs of patients with DRGs through compliance.

**Table 4:** The adaptation to DRGs payment reform.

Variables	*N*	%
The self-perception of adaptation to reform (Subjectivity)		
How adaptable are you to the DRGs payment reform?		
Completely maladaptive	18	2.9
Maladaptive	75	11.9
Average	375	59.5
Adaptive	153	24.3
Completely adaptive	9	1.4
The adaptive behaviors to reform (Objectivity)		
Will you take the initiative to pay attention to and control the medical expenses of DRGs patients by standardizing their diagnosis and treatment?		
Never	32	5.1
Occasionally	250	39.7
Offen	268	42.5
Every case	80	12.7
Will you actively participate in the management of DRGs patients through standardizing the writing of medical records, analyzing cost data, learning medical insurance policies, improving hospital operations, and so on?		
Never	37	5.9
Occasionally	274	43.5
Offen	276	43.8
Every case	43	6.8

### 4.4 Cognition of DRGs payment policy

As shown in Table 5, according to the scores of the respondents, their knowledge level of DRGs payment policy was generally medium to above, with a relatively high understanding of basic knowledge, but an average understanding of more specific rules and systems. Meanwhile, their identity of the instrumental value, economic value, and social value of the DRGs payment policy was average.

**Table 5:** The policy cognition scores of medical staff.

Variables	M (P25, P75)
Policy awareness	
The concept of DRGs	4 (3,4)
The calculation methods of weights and rates in DRGs	3 (3,4)
The meaning of Case Mix Index (CMI)	3 (3,4)
The violations stipulated in DRGs payment supervision (Such as turning away critically ill patients, disaggregating hospitalization costs, discharging the patient early, setting up disease groups with higher points, etc.)	3 (2,4)
Medical insurance compensation policy for DRGs cases using clinical new technologies (Such as Da Vinci robot, femtosecond, TAVI, etc.)	4 (3,4)
Medical insurance compensation policy for DRGs cases using nationally negotiated medicine	3 (3,4)
Policy identity	
Standardizing diagnosis, treatment, and charging behaviors	3 (3,4)
Scientific response to disease difficulty difference	3 (3,4)
Promoting the development of the discipline	3 (3,4)
Promoting the development of new medical technologies	3 (2,4)
Reducing the financial burden of patients	3 (2,4)
Improving the quality of medical services	3 (3,4)
Improving the efficiency of medical services	3 (2,3)
Promoting hierarchical diagnosis and treatment	3 (2,3)

### 4.5 Pre-test results

Under the condition of controlling age, gender, position, and other basic characteristic factors, the results of partial correlation regression analysis among core variables showed that there was a certain correlation between them, as shown in Table 6. Subsequently, the core variables were included in the multiple linear regression model successively according to the research requirements, and the *F*-test results all showed that the model had statistical significance as a whole (*p* < 0.05), and the VIF value of each variable was less than 10, which proved that there was no collinearity problem.

**Table 6:** Correlation analysis of the core variables.

Partial correlation coefficient (*r*)	Y_1_	Y_2_	X_1_	X_2_	X_3_	M_1_	M_2_
Y_1_	1.000^**^	0.241^**^	0.188^**^	0.357^**^	0.365^**^	0.417^**^	0.390^**^
Y_2_	0.241^**^	1.000^**^	0.305^**^	0.290^**^	0.244^**^	0.384^**^	0.149^**^
X_1_	0.188^**^	0.305^**^	1.000^**^	0.251^**^	0.234^**^	0.435^**^	0.077
X_2_	0.357^**^	0.290^**^	0.251^**^	1.000^**^	0.659^**^	0.294^**^	0.352^**^
X_3_	0.365^**^	0.244^**^	0.234^**^	0.659^**^	1.000^**^	0.302^**^	0.339^**^
M_1_	0.417^**^	0.384^**^	0.435^**^	0.294^**^	0.302^**^	1.000^**^	0.234^**^
M_2_	0.390^**^	0.149^**^	0.077^*^	0.352^**^	0.339^**^	0.234^**^	1.000^**^

** and * represented the significance level of 1 and 5%, respectively.

### 4.6 Intermediate effect test

**Figure 2:**
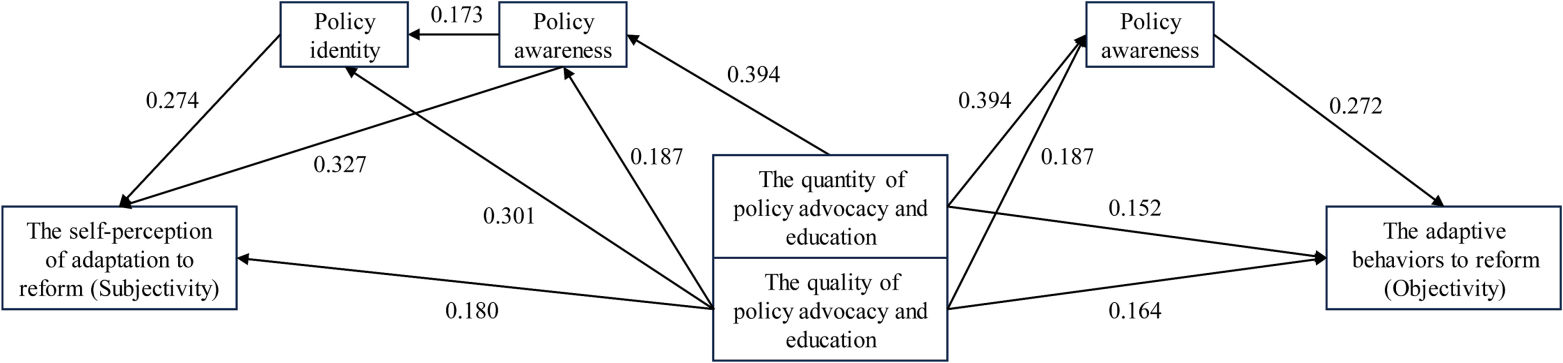
Mediation effect paths diagram (Only significant paths were displayed).

Combined with the analysis results in Tables 7, 8, it was shown that policy cognition had seven effective mediating paths in the process of policy advocacy and education promoting medical staff to adapt to the DRGs payment reform because the 95% confidence interval of Bootstrap sampling of their mediating effect value did not contain zero. These pathways included both parallel mediation and chain mediation, as well as partial mediation and complete mediation, among which the largest mediation effect value was 0.129, and the proportion of its effect (the ratio of mediating effect to total effect) was 87.2%, and the total mediation effect value of policy cognition was 0.148, 0.152, 0.108, and 0.057, respectively. At the same time, we found that the mediating effect of policy awareness was relatively larger than that of policy identity, and the chain mediating effect formed by the two was relatively smaller than that of the separate parallel mediating effect. Therefore, this study also drew the mediating effect paths diagram to better display and understand the research results, as shown in Figure 2.

**Table 7:** The model testing of the mediation effects.

Variables	Model 1 (Y1)	Model 2 (Y2)	Model 3 (M1)	Model 4 (M2)	Model 5 (Y1)	Model 6 (Y2)
Sex	−0.083 (−1.821)	−0.027 (−0.618)	−0.117^**^ (−2.881)	−0.012 (−0.273)	−0.036 (−0.859)	0.005 (0.127)
Age	0.173 (1.167)	−0.153 (−1.072)	−0.143 (−1.077)	−0.058 (−0.421)	0.242 (1.796)	−0.113 (−0.815)
Education	−0.124^*^ (−1.989)	−0.02 (−0.369)	0.080 (1.429)	−0.200^**^ (−3.451)	−0.099 (−1.730)	−0.041 (−0.694)
Occupation type	0.138^*^ (2.261)	−0.163^**^ (−2.769)	−0.042 (−0.771)	0.257^**^ (4.536)	0.084 (1.479)	−0.155^**^ (−2.685)
Positional title	−0.033 (−0.559)	0.065 (1.142)	−0.004 (−0.067)	−0.045 (−0.812)	−0.020 (−0.363)	0.067 (1.210)
Years of service	−0.152 (−1.026)	0.176 (1.236)	0.205 (1.543)	−0.051 (−0.371)	−0.215 (−1.591)	0.121 (0.873)
X_1_	0.114^**^ (2.743)	0.259^**^ (6.468)	0.394^**^ (10.565)	−0.081 (−1.919)	−0.011 (−0.271)	0.152^**^ (3.601)
X_2_	0.332^**^ (8.581)	0.221^**^ (5.924)	0.187^**^ (5.410)	0.301^**^ (8.178)	0.180^**^ (4.743)	0.164^**^ (4.225)
M_1_				0.173^**^ (4.145)	0.327^**^ (7.922)	0.272^**^ (6.431)
M_2_					0.274^**^ (6.960)	0.017 (0.411)
*R*^2^	0.156	0.220	0.325	0.275	0.305	0.272
Adjusted *R*^2^	0.145	0.210	0.316	0.265	0.294	0.260
*F*	14.344^**^	21.931^**^	37.360^**^	26.146^**^	27.182^**^	23.076^**^

The table included standardized regression coefficients and *t* values in parentheses. ** and * represented the significance level of 1 and 5%, respectively,.

**Table 8:** The results of mediation effect analysis.

Terms	Effect	Boot SE	BootLLCI	BootULCI	Types of mediating effect	Proportion of mediating effect
X_1_ → M_1_ → Y_1_	0.129	0.021	0.090	0.174	Parallel mediation	87.2%
X_1_ → M_2_ → Y_1_	−0.022	0.014	−0.054	0.001	Insignificant effect	0.0%
X_1_ → M_1_ → M_2_ → Y_1_	0.019	0.006	0.009	0.032	Chain mediation	12.8%
X_2_ → M_1_ → Y_1_	0.061	0.015	0.035	0.095	Parallel mediation	18.4%
X_2_ → M_2_ → Y_1_	0.082	0.017	0.052	0.120	Parallel mediation	24.7%
X_2_ → M_1_ → M_2_ → Y_1_	0.009	0.003	0.004	0.017	Chain mediation	2.7%
X_1_ → M_1_ → Y_2_	0.107	0.022	0.069	0.157	Parallel mediation	41.3%
X_1_ → M_2_ → Y_2_	−0.001	0.004	−0.012	0.005	Insignificant effect	0.0%
X_1_ → M_1_ → M_2_ → Y_2_	0.001	0.003	−0.005	0.007	Insignificant effect	0.0%
X_2_ → M_1_ → Y_2_	0.051	0.013	0.028	0.078	Parallel mediation	23.1%
X_2_ → M_2_ → Y_2_	0.005	0.013	−0.022	0.028	Insignificant effect	0.0%
X_2_ → M_1_ → M_2_ → Y_2_	0.001	0.001	−0.002	0.004	Insignificant effect	0.0%

SE represented standard error, BootLLCI and BootULCI referred to the lower and upper limits of the 95% confidence interval of the Bootstrap sampling.

### 4.7 Sensitivity analysis

To ensure the reliability of the research results, we applied the variable substitution method to carry out the sensitivity analysis, that is, the independent variable X_2_ was replaced by X_3_, and the intermediary effect analysis was carried out according to the steps above. The results were shown in Tables 9, 10, and the mediating action path, effect value, and effect proportion had basically not changed, which indicated that the research results had a certain credibility.

**Table 9:** Sensitivity analysis of the models in Table 7.

Variables	Model 1 (Y_1_)	Model 2 (Y_2_)	Model 3 (M_1_)	Model 4 (M_2_)	Model 5 (Y_1_)	Model 6 (Y_2_)
Sex	−0.070 (−1.534)	−0.021 (−0.465)	−0.110^**^ (−2.696)	−0.001 (−0.022)	−0.029 (−0.700)	0.011 (0.247)
Age	0.207 (1.405)	−0.128 (−0.887)	−0.124 (−0.938)	−0.026 (−0.186)	0.260 (1.934)	−0.091 (−0.658)
Education	−0.102 (−1.641)	−0.012 (−0.190)	0.093 (1.667)	−0.182^**^ (−3.117)	−0.086 (−1.510)	−0.031 (−0.530)
Occupation type	0.155^*^ (2.541)	−0.155 (−2.606)	−0.032 (−0.592)	0.271^**^ (4.743)	0.093 (1.639)	−0.155^**^ (−2.658)
Positional title	−0.013 (−0.220)	0.076 (1.329)	0.008 (0.154)	−0.027 (−0.496)	−0.008 (−0.158)	0.075 (1.352)
Years of service	−0.188 (−1.272)	0.153 (1.059)	0.185 (1.397)	−0.083 (−0.601)	−0.233 (−1.730)	0.103 (0.739)
X_1_	0.118^**^ (2.854)	0.274^**^ (6.803)	0.394^**^ (10.651)	−0.070 (−1.669)	−0.008 (−0.190)	0.164^**^ (3.878)
X_3_	0.340^**^ (8.894)	0.176^**^ (4.731)	0.200^**^ (5.841)	0.282^**^ (7.684)	0.189^**^ (5.055)	0.109^**^ (2.824)
M_1_				0.171^**^ (4.071)	0.321^**^ (7.767)	0.278^**^ (6.503)
M_2_					0.274^**^ (7.018)	0.036 (0.893)
*R*^2^	0.163	0.205	0.330	0.267	0.308	0.260
Adjusted *R*^2^	0.152	0.195	0.321	0.256	0.297	0.248
*F*	15.066^**^	20.003^**^	38.218^**^	25.062^**^	27.606^**^	21.757^**^

The table included standardized regression coefficients and *t* values in parentheses, ** and * represented the significance level of 1 and 5%, respectively.

**Table 10:** Sensitivity analysis of the mediation effect test results.

Terms	Effect	Boot SE	BootLLCI	BootULCI	Types of mediating effect	Proportion of mediating effect
X_1_ → M_1_ → Y_1_	0.127	0.022	0.087	0.173	Parallel mediation	87.6%
X_1_ → M_2_ → Y_1_	−0.019	0.013	−0.050	0.004	Insignificant effect	0.0%
X_1_ → M_1_ → M_2_ → Y_1_	0.018	0.006	0.009	0.032	Chain mediation	12.4%
X_3_ → M_1_ → Y_1_	0.064	0.014	0.040	0.096	Parallel mediation	18.8%
X_3_ → M_2_ → Y_1_	0.077	0.018	0.047	0.116	Parallel mediation	22.6%
X_3_ → M_1_ → M_2_ → Y_1_	0.009	0.003	0.004	0.018	Chain mediation	2.6%
X_1_ → M_1_ → Y_2_	0.110	0.022	0.073	0.160	Parallel mediation	40.1%
X_1_ → M_2_ → Y_2_	−0.003	0.004	−0.016	0.002	Insignificant effect	0.0%
X_1_ → M_1_ → M_2_ → Y_2_	0.002	0.003	−0.003	0.009	Insignificant effect	0.0%
X_3_ → M_1_ → Y_2_	0.056	0.013	0.032	0.082	Parallel mediation	31.8%
X_3_ → M_2_ → Y_2_	0.010	0.012	−0.013	0.034	Insignificant effect	0.0%
X_3_ → M_1_ → M_2_ → Y_2_	0.001	0.002	−0.001	0.005	Insignificant effect	0.0%

SE represented standard error, BootLLCI and BootULCI referred to the lower and upper limits of the 95% confidence interval of the Bootstrap sampling.

## 5 Discussion

The research results showed that organized and focused policy advocacy and education could promote the adaptation of medical staff to the DRGs payment reform by improving their cognition of the policy. Although this conclusion was the first to be proposed, many similar research conclusions that could be used as evidence. On the one hand, many practices and studies have confirmed that policy advocacy and education have an effective effect on people’s cognition and behavior, especially in the field of health intervention of health policy (40, 41). In addition, Doaty, Sarah and others also put forward more specific advocacy and education action plans (45). On the other hand, a large number of studies have shown that policy cognition could have an important impact on the public’s behavioral intention and results, including consumption behavior, health behavior, production behavior, and other fields (49–52). In addition, Zhou, Lingyi, Beasley, Lisa, and others also conducted a series of studies on the connotation of policy cognition and how it affected the adaptation to reform (38, 43). On this basis, this study suggested that it was no accident that policy advocacy and education could help medical staff adapt to health insurance reforms. This was because medical staff, although they were direct stakeholders in the DRGs payment reform, had a greater information asymmetry compared with policymakers, which made the enforcement of reform and their inherent cognition and behavior habits have a fierce collision, the phenomenon of maladaptation or superficial adaptation brought about by passive obedience was widespread (13, 21, 24). However, timely and accurate policy advocacy and education could just make up for their cognitive deficiencies, reduce misunderstanding and even resistance, and promote them to make correct behavioral responses to achieve real adaptation (44).

At the same time, we found that the mediating effects of policy awareness and policy identity were both chain and parallel, that is, there were ideal mediating paths from policy awareness to policy identity and non-ideal paths that only directly affected through policy awareness or policy identity, and the latter effect accounted for a relatively large proportion. The diversity of intermediary paths showed the complexity of policy advocacy and education influence process and also indicated that there were some problems on its own (43). The ideal action path conforms to both the knowledge-attitude-practice theory and the process of policy acceptance response from low to high, but the action path lacking policy awareness or policy identity often represents blind identification or passive obedience (25, 39), which belongs to the surface adaptation state and is not stable. Although this phenomenon is related to the differences in cognitive ability and social environment of medical staff (53), the universality and pertinence of policy advocacy and education still cannot be ignored.

The results also showed that the more times the medical staff participated in the policy advocacy and education activities or the better the feedback evaluation of them could promote their adaptation to the reform. However, there were differences in their action paths. The former must be based on improving the level of policy awareness to have an impact, and the overall effect of paths was relatively large. The latter could directly influence the adaptation to reform through various paths, but the overall effect is relatively small. This indicated that in the early stage of the reform, the information gap among medical staff was large, and a lot of policy advocacy and education could quickly improve their cognition, and then promote their response to new things, and finally achieve the state of adaptation. It is worth noting that this process of causing qualitative change through quantitative change will consume a lot of time so that the intervention efficiency is low (54). Therefore, improving the quality of advocacy and education is the long-term means to promote the adaptation to reform, which requires the organizers to pay attention to the feedback of medical staff promptly, formulate targeted advocacy and education strategies that meet the needs, and enrich the value connotation of it, so as to play a positive role in the cultivation of medical staff (55, 56).

In addition, we also found that medical staff’s self-perception and behavioral performance of adaptive state were affected by different paths, the former had a more extensive influence path and relatively large mediating effect, while the latter only promoted the generation of adaptive behaviors by improving the level of policy awareness. This indicated that the influence of policy advocacy and education had not realized the ideal mechanism of the knowledge-attitude-practice theory among the respondents, which once again confirmed the existence of the phenomenon of passive obedience. It is not hard to understand, on the one hand, due to the compulsory and dynamic nature of the reform, medical staff had to make adaptive responses in a short period to reduce conflicts of interest, such as choosing more cost-effective diagnosis and treatment plans, regulating fees, writing medical records more carefully, and introducing DRGs-related indicators for performance assessment, which conflicted with their perception under the old medicare payment system (29, 57). On the other hand, the new payment method itself was not perfect, such as unreasonable disease grouping, unscientific calculation method of payment standard, imperfect compensation mechanism, etc. (58, 59), coupled with insufficient policy advocacy and education, medical staff did not agree with the DRGs payment reform, and even conflict, thus forming a surface adaptation state with only objective behavior but no subjective perception.

## 6 Limitations

Firstly, although the questionnaire has passed the reliability and validity test, the design of the questionnaire is still subjective and has not been widely used before, and its universality needs to be further verified. Secondly, the sampling scope of this study is limited, and the sampling method is only approximate stratified sampling and convenience sampling, which is not completely random, which will cause some confusion or bias in the research results. In addition, due to the fact that there are currently few literatures related to the content of this study, although the innovativeness of the article is proved, the discussion section has relatively little literature basis.

## 7 Practical implications and theoretical implications

In terms of practical implications, this study provided a basis for policymakers and hospital managers to develop interventions to promote medical staff to adapt to DRGs payment reform, including constantly improving the DRGs payment system, increasing the number of organized collective policy advocacy and education, paying attention to the needs and feedback of medical staff, formulating targeted, timely and accurate policy advocacy and education content, highlighting the value connotation of DRGs payment reform, etc.

In terms of theoretical implications, this study combined the multidisciplinary theory to define the state of adaptation to reform, and designed the survey dimensions and questions accordingly, which provided a framework for investigation and research in related fields. Meanwhile, this study also revealed the complexity of the influence path of policy advocacy and education on cognition and behavior, and the process was affected by multiple factors, which did not necessarily fully conform to the traditional theory of knowledge-attitude-practice theory.

## 8 Conclusion and suggestion

Policy advocacy and education can promote medical staff’s adaptation to DRGs payment reform by improving their policy cognition, and the action paths are diverse. Policymakers and hospital managers should pay attention to this phenomenon, and formulate demand-centered, value-oriented whole-process advocacy and education strategies while constantly improving the DRGs payment system, so as to enable medical staff to adapt to the current reform faster and better. These also provided a basis for further research and practice of positive intervention in DRGs payment reform.

## Data Availability

The raw data supporting the conclusions of this article will be made available by the authors, without undue reservation.
