# Supplementary figures and images for: The influence of policy advocacy and education on medical staff’s adaptation to diagnosis related groups payment reform in China: an analysis of the mediating effect of policy cognition
Source: Front Public Health. 2024 Nov 13;12:1375739. doi: 10.3389/fpubh.2024.1375739 (PMC11599170; doi:10.3389/fpubh.2024.1375739)

**
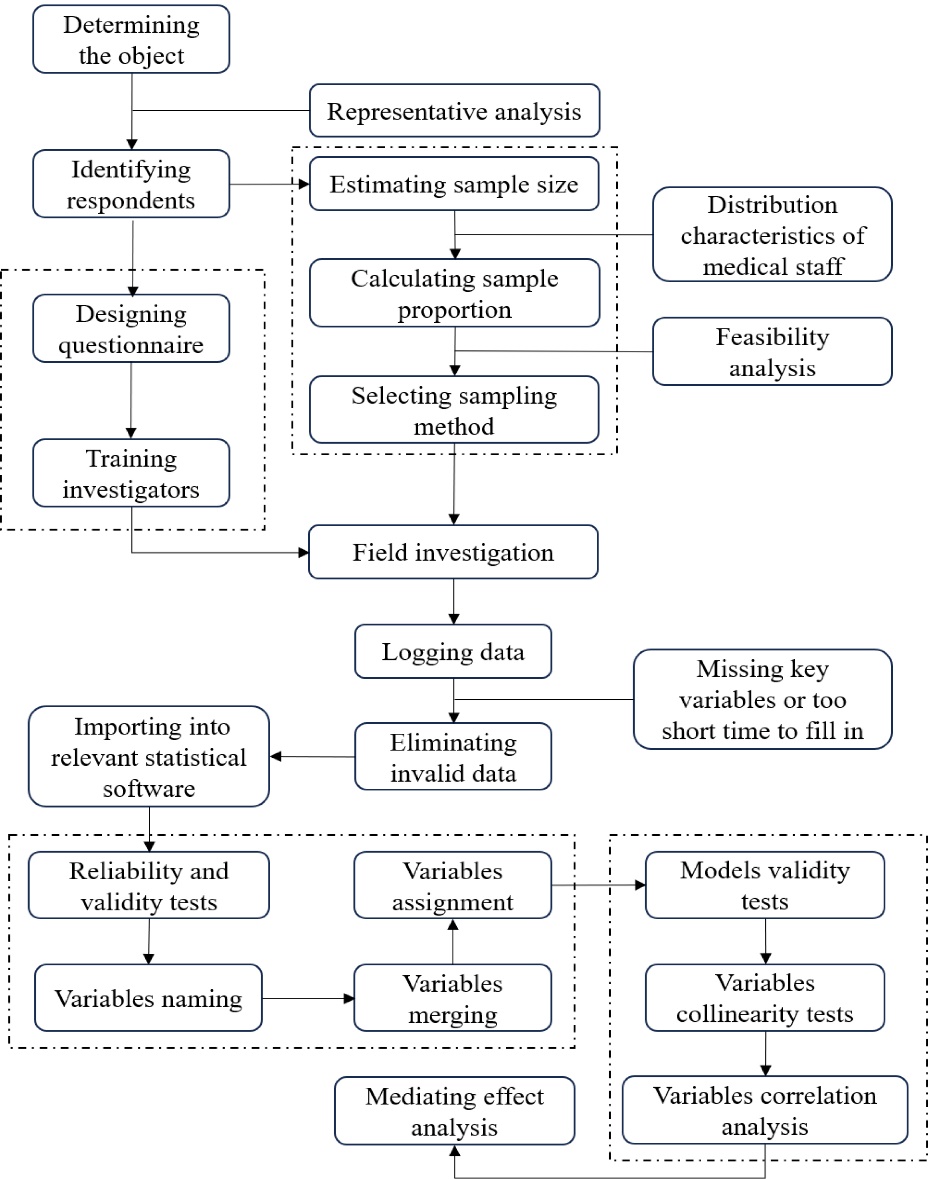
**

**Supplementary Figure 1. The data collection and processing program diagram.**

Supplement: Supplementary file 1 [file Supplementary_file_1.docx]
