# Supplementary material for: The influence of policy advocacy and education on medical staff’s adaptation to diagnosis related groups payment reform in China: an analysis of the mediating effect of policy cognition
Source: Front Public Health. 2024 Nov 13;12:1375739. doi: 10.3389/fpubh.2024.1375739 (PMC11599170; doi:10.3389/fpubh.2024.1375739)
Supplement: Supplementary file 2 [file Supplementary_file_2.docx]

***Appendix***

**Questionnaire**

**Ⅰ Basic information**

1. Sex: A. Male B. Female

2. Age: years old

3. Education: A. Doctor's degree B. Master's degree

C. Bachelor's degree or below

4. Years of service: years

5. Occupation type: A. Doctor B. Medical technician C. Nurse

6. Positional title:  A. Senior B. Intermediate C. Primary or below

**Ⅱ The cognition of DRGs payment policy**

1. How much do you know about the following relevant contents of DRGs payment policy? (A higher score indicates a higher level of awareness)

| **Items** | **Scores (1-5)** |
| --- | --- |
| The concept of DRGs |  |
| The calculation methods of weights and rates in DRGs |  |
| The meaning of Case Mix Index (CMI) |  |
| The violations stipulated in DRGs payment supervision (Such as turning away critically ill patients, disaggregating hospitalization costs, discharging the patient early, setting up disease groups with higher points, etc) |  |
| Medical insurance compensation policy for DRGs cases using clinical new technologies (Such as Da Vinci robot, femtosecond, TAVI, etc) |  |
| Medical insurance compensation policy for DRGs cases using nationally negotiated medicine |  |

1. What do you think of the role of the following DRGs payment policy? (The higher the score, the greater the effect)

| **Items** | **Scores (1-5)** |
| --- | --- |
| Standardizing diagnosis, treatment, and charging behaviors |  |
| Scientific response to disease difficulty difference |  |
| Promoting the development of the discipline |  |
| Promoting the development of new medical technologies |  |
| Reducing the financial burden of patients |  |
| Improving the quality of medical services |  |
| Improving the efficiency of medical services |  |
| Promoting hierarchical diagnosis and treatment |  |

**Ⅲ The policy advocacy and education about DRGs payment**

1. Where did you learn about the information about DRGs payment policy? (Multiple choice)

A. Training or meetings conducted by the hospital medical insurance office

B. Lectures organized by the superior medical insurance department

C. Network platform

D. TV, radio, newspapers, and other traditional media

E. Relatives or friends

F. Other:

1. How many times have you received organized collective training, lectures, or meetings on the DRGs payment policy in the past year? (Informal or individual learning activities are not included)

A. 0 B. 1 C. 2 D. 3 E. 4 F. ≥5

1. Do you think it is helpful for you to understand and adapt to the DRGs payment reform after attending the above activities?

A. Not helpful at all

B. Less helpful

C. Average

D. Relatively helpful

E. Very helpful

1. What is your overall satisfaction with the above activities you have participated in?

A. Very dissatisfied

B. Somewhat dissatisfied

C. Average

D. Somewhat satisfied

E. Very satisfied

1. How often do you think DRGs payment policy advocacy and education should be conducted?

A. Every month

B. Every quarter

C. Every half year

D. Every year,

E. Longer

1. What DRGs payment policy advocacy and education content are you more interested in? (Multiple choice)

A. Common sense of policy

B. Code of conduct for diagnosis, treatment, charging and writing medical records

C. Use of management system

D. Data analysis

E. Other:

**Ⅳ The adaptation to DRGs payment reform**

1. How adaptable are you to the DRGs payment reform?

A. Completely maladaptive

B. Maladaptive

C. Average

D. Adaptive

E. Completely adaptive

1. Will you take the initiative to pay attention to and control the medical expenses of DRGs patients by standardizing their diagnosis and treatment?

A. Never

B. Occasionally

C. Often

D. Every case

1. Will you actively participate in the management of DRGs patients through standardizing the writing of medical records, analyzing cost data, learning medical insurance policies, improving hospital operations, and so on?

A. Neve

B. Occasionally

C. Often

D. Every case
